# Factors associated with quality of life among chronic kidney disease patients in Nepal: a cross-sectional study

**DOI:** 10.1186/s12955-020-01458-1

**Published:** 2020-06-29

**Authors:** Shambhu Kumar Saxena Mahato, Tawatchai Apidechkul, Pamornsri Sriwongpan, Rajani Hada, Guna Nidhi Sharma, Shravan Kumar Nayak, Ram Kumar Mahato

**Affiliations:** 1 School of Health Science, Mae Fah Luang University, Muang Chiang Rai, Chiang Rai Province Thailand; 2 Epidemiology and Disease Control Division, Department of Health Services, Teku, Kathmandu, Nepal; 3 Center of Excellence for the Hill tribe Health Research, Mae Fah Luang University, Muang Chiang Rai, Chiang Rai Province Thailand; 4 Department of Nephrology, National Academy of Health Sciences, Bir Hospital, Mahaboudh, Kathmandu, Nepal; 5 Ministry of Health and Population, Ramshah Path, Kathmandu, Nepal; 6 Ministry of Social Development, Province Number 2, Janakpurdham, Nepal

**Keywords:** CKD patients, Factors associated, Good-quality of life, Nepal, Prevalence

## Abstract

**Background:**

Chronic kidney disease (CKD) leads to decreased quality of life (QOL) by increasing the risk of death during the progression of its pathogenesis. However, many factors can be improved to support QOL. This study aimed to assess QOL among CKD patients in Nepal and to determine the factors associated with their QOL.

**Method:**

A cross-sectional study was used for data collection. CKD cases receiving medical attention in the Bir Hospital in Mahaboudh, Kathmandu; Tribhuvan University Teaching Hospital in Maharajgunj, Kathmandu; Sumeru Hospital in Dhapakhel, Lalitpur; and Shahid Dharma Bhakta National Transplant Centre in Bhaktapur between August and October 2019 were invited to participate in the study. A validated questionnaire and the kidney disease quality of life short form (KDQOL-SF™ 1.3) were used to assess QOL. A questionnaire was completed by the researcher in face-to-face interviews. Logistic regression was used to detect the associations between variables at the significance level of α = 0.05.

**Results:**

A total of 440 participants were recruited into the study: 56.59% were males, 74.32% were aged between 31 and 70 years, 25.68% were illiterate, and 82.95% were unemployed. The prevalence of good QOL among CKD in the domains of the physical component summary (PCS), mental component summary (MCS), and kidney disease component summary (KDCS) with and without hemodialysis were 53.64, 22.05, 21.28, and 13.19%, respectively. After controlling for all potential confounding factors, eight variables were found to be associated with good QOL in the domain of PCS: age, education, stage of CKD, hemodialysis, transporting oneself to a hospital, health insurance, medical expenses, and perceived lack of difficulty in handling medical expenses. Six variables were associated with good QOL in the domain of MCS after controlling for all potential confounding factors: residence, stage of CKD, transporting oneself to a hospital, health insurance, medical expenses, and perceived lack of difficulty in handling medical expenses.

**Conclusions:**

Public health interventions should be developed and implemented to improve QOL among CKD patients in Nepal by focusing on older female patients who have low education, live in rural areas and no health insurance.

## Background

Chronic kidney disease (CKD) has become a global burden on the health service system [1] and has been recognized as a major threat to humans, particularly in reducing quality of life (QOL) in the later stages of the disease [2]. Most CKD patients are reported to be in middle-aged and elderly populations [3] and are clearly found in both developed and developing countries [4]. A large proportion of CKD cases are diagnosed with other chronic noncommunicable diseases, such as hypertension and diabetes mellitus [5]. CKD impacts several dimensions, such as individual quality of life [6], family income [7], and reduction in contributions to social [8] and national development [9]. Moreover, CKD patients have become major consumers of public health resources, particularly in hemodialysis clinics [10]. In 2015, there were 1.1 million deaths worldwide due to CKD [11], which was the 12th leading cause of death [11]. In 2017, 1,230,200 people died due to CKD-related causes worldwide, which represented a 33.70% increase in the death rate for the period of 2007–2017 [12]. Within the domains of basic human rights and the right to access healthcare, individuals want to obtain better physical and mental health, have a longer life, and experience improved quality of life through different interventions. These demands have been dramatically increasing worldwide, including in Nepal [13].

Nepal is located in Southeast Asia and has an approximate total population of 28 million people [14]. In general, individuals who receive a medical treatment or service are required to pay out of pocket [15, 16]. However, some chronic diseases, including CKD, are covered by medical insurance when patients receive services in some hospitals [17]. Today, all CKD patients are provided with hemodialysis free of charge in 55 hospitals that are supported directly by the government [18]. The hospitals that provide free-of-charge hemodialysis are distributed throughout the country. Today, approximately 3,000,000 CKD patients attend clinics both with and without hemodialysis services [18].

Nepal had a GDP of 24.47 billion in 2017 [19] and was classified economically as a low-income country [20]. Due to the nature of CKD, most patients need to access hemodialysis, particularly in the later stage of the disease [21]. The frequency and accessibility of hemodialysis depend on the patient’s profile and availability of quality healthcare services [22]. However, most CKD patients in Nepal are able to access some medical services [18]. Due to the accessibility of medical care and the attention paid by the government to this illness, a large proportion of CKD patients have a longer life compared with previous eras [23]. Improving the QOL of CKD patients has become a key issue for the government and healthcare providers [24]. There are several impacts on patients who develop CKD, including a reduction in their QOL [25]. All CKD patients want to have a longer life with a better QOL. In general, several different factors influence QOL for a population or a specific patient. Therefore, investigating the QOL levels and factors associated with QOL among CKD patients, particularly those living in constrained economies such as in Nepal, is a highly important issue. There might be improvements in the quality of health services and collaborations among stakeholders.

This study aimed to assess QOL and determine the factors associated with QOL among CKD patients in Nepal by using the standard kidney disease quality of life short form (KDQOL-SF™ 1) [26] in the domains of the physical component summary (PCS) and mental component summary (MCS).

## Methods

### Study design

A hospital-based cross-sectional study aimed to assess QOL among CKD patients in Nepal and to determine the factors associated with good QOL among CKD patients.

### Study setting

The study was conducted in 4 of 6 hospitals in Kathmandu Valley that provide free treatment and hemodialysis for CKD patients [18]: Bir Hospital in Mahaboudh, Kathmandu; Tribhuvan University Teaching Hospital in Maharajgunj; Kathmandu, Sumeru Hospital in Dhapakhel, Lalitpur; and Shahid Dharma Bhakta National Transplant Centre in Bhaktapur. Bir Hospital, Tribhuvan University Teaching Hospital, and Shahid Dharma Bhakta National Transplant Centre are tertiary-level public hospitals, but Sumeru Hospital is a private hospital. Bir Hospital and Tribhuvan University Teaching Hospital are located in the Kathmandu District, Shahid Dharma Bhakta National Transplant Centre is located in the Bhaktapur District, and Sumeru Hospital is located in the Lalitpur District, Bagmati Province, Nepal.

### Study population

The study population comprised all stages of CKD patients aged 18 years and over living in Kathmandu Valley.

### Eligible population

The inclusion criteria for this study were CKD patients aged 18 years and over who had a confirmed diagnosis of CKD by a medical doctor who attended the selected hospitals: Bir Hospital in Mahaboudh, Kathmandu; Tribhuvan University Teaching Hospital in Maharajgunj, Kathmandu’ Sumeru Hospital in Dhapakhel, Lalitpur’ and Shahid Dharma Bhakta National Transplant Centre in Bhaktapur from September to November 2019. Those who could not provide all the essential information on the questionnaire were excluded from the study.

### Sample size

The sample size was calculated by the standard formula of a cross-sectional study [27], where Z *α*/_2_ = 1.96; e = 0.05; p = estimated proportion of the prevalence of good QOL in CKD patients in Nepal, which was 30.00% = 0.30 [18]; and q = 0.70. At least 323 participants were needed for the analysis. After adding 20.0% to account for any errors in the study process, 387 participants were required for the study.

### Research instruments

A validated questionnaire and standard tool were used for data collection. The validated questionnaire was developed with insights from a review of several articles related to this study and with professional support from experts in the fields.

The validated questionnaire consisted of 23 questions in four parts. In part one, nine questions were used to collect the general characteristics of the participants: age, sex, educational level, occupational status, household income, marital status, ethnicity, religion, and place of residence. In part two, five questions were used to collect the patient’s stage of CKD and experience of treatment: duration of illness, stage of CKD, whether the patient is receiving hemodialysis, duration of hemodialysis, and transplantation history. In part three, three questions were used to collect information on support from various populations: having been taken to a hospital, support for medical expenses, and having health insurance. In part four, six questions were used to collect the burden, impacts, and complications due to CKD: frequency of seeing a doctor per month, distance from the hospital, medical expenses per visit, problems paying medical expenses, coexistence of NCDs, and disabilities.

The standard tool for detecting quality of life among CKD patients (revised version 1.3 KDQOL-SF™) [26] was used to assess the quality of life of the participants. KDQOL-SF includes 24 questions for assessing QOL in 3 domains: PCS (physical component summary), MCS (mental component summary), and KDCS (kidney disease component summary). In domain one, 21 questions were used to assess QOL in the PCS domain: 10 for physical function, 4 for role function, 2 for pain, and 5 for general health-related questions. In domain two, 14 questions were used to assess QOL in the MCS domain: 5 for the physical role, 3 for the emotional role, 2 for social function, and 4 for energy/fatigue-related questions. In domain three, 43 questions were used to assess QOL in KDCS domains: 12 for symptoms, 8 for effects, 4 for burden of kidney disease, 2 for work status, 3 for cognitive function, 3 for quality of social interactions, 2 for sexual function, 4 for sleep, 2 for social support, 2 for dialysis staff encouragement, and 1 for a patient satisfaction-related question.

The mean and SD in each domain were used to divide the level of QOL (according to the guidelines of KDQOL COMPLETE) [28] into three levels: poor QOL, moderate QOL, and good QOL. For poor QOL, the level was less than the mean-1 SD; for moderate QOL, the level was equal to the mean +/− SD; and for good QOL, the level was more than the mean + 1 SD.

The research instrument was developed in English and then translated into Nepali and back into English before use for data collection.

### Research instrument development

For the questionnaire, the item objective congruence (IOC) method was used to improve the content validity by three external experts: 2 public health experts and one medical doctor who worked in the field. The experts were selected due to their experience in clinical work with CKD patients and their experience in conducting research in the field of QOL. Each expert provided a score for each item with comments: 1 for clearly relevant to the study, − 1 for clearly not relevant to the study, and 0 for the content area is unclear regarding a connection with the study. For the evaluation results, any question that scored ≤0.5 was deleted from the questionnaire, any question that scored 0.5–0.7 was revised according to the comments, and any question that scored > 0.7 was included in the questionnaire.

For reliability detection, the questionnaire was developed in English, translated into Nepali and back translated from Nepali into English. Translation was performed by three Nepalese who were fluent in both English and Nepali. Before data collection, a pilot test was performed in the National Kidney Center in Bhairab Bhawan, Kathmandu, among 30 sample patients who had similar characteristics to the study sample. The reliability, feasibility and ordering of the questions were determined. The reliability presented with Cronbach’s alpha =0.74, which was acceptable for the study.

### Steps of data collection

Permission to access the hospitals was granted by the hospital directors. Then, the chiefs of the nephrology units were contacted and received detailed explanations about the study protocols. Appointments for data collection were made at least two weeks in advance. At the date of data collection, all participants were invited to participate in the study and to provide information after completion of their hemodialysis or after they met with a doctor. All patients attending the selected hospitals who met the criteria were invited to answer the questionnaire during September–November 2019. Participants were given all the essential information regarding the study and provided written consent before starting the interview. The questionnaire was completed by a researcher who was fluent in Nepali during face-to-face interviews, each of which lasted for approximately 20 min.

### Statistical analysis

Data were coded and double entered into an Excel sheet before being transferred into SPSS version 18 (SPSS, Chicago, IL) for analysis. Categorical data are presented as percentages. Continuous data, the means and SDs, were described for normal distributions, and medians and IQIs were described for non-normal distributions. Simple (univariate) and multiple (multivariate) logistic regressions were used to detect the associations between variables at a significance level at α = 0.05. The mode of “Enter” was used to select the variable into the model in both the simple and multiple analyses based on the conceptual framework of the study. All the variables were found to be significant in the univariate analysis and were then input into the model for multivariate analysis. To find the best model, the pseudo R-squared test and Hosmer-Lemeshow chi-square test were used to consider the fit of the model before interpreting the final model.

## Results

Four hundred-forty participants were invited to participate in the study; 56.59% were male, and their mean of the age was 52.73 years (SD = 15.96, min = 19, and max = 84). Three-fourths (76.82%) of participants were married, 25.68% were illiterate, and 82.95% were unemployed. Three hundred thirty-three participants were Hindus, 40.22% were from the Janjati ethnic group, and 62.95% were living in a village as their place of residence (Table 1).

**Table 1:** General characteristics of participants

Characteristics	n	%
**Total**	**440**	**100.0**
**Sex**		
Male	249	56.59
Female	191	43.41
**Age** (year)		
< 31	58	13.18
31–50	142	32.27
51–70	185	42.05
> 70	55	12.50
Min. = 19, Max. = 84, Mean = 52.73, SD = 15.96		
**Education**		
Illiterate	113	25.68
Primary	99	22.50
Secondary	168	38.18
Tertiary	60	13.64
**Occupation**		
Employed	30	6.82
Retired	45	10.23
Unemployed	365	82.95
**Annual household income** (NRs^a^)		
No income	90	20.45
< 1,00,000	209	47.50
1,00,000-5,00,000	108	24.55
6,00,000-10,00,000	33	7.50
**Marital status**		
Single	47	10.68
Married	338	76.82
Widow/widower	36	8.18
Divorced or separated	19	4.32
**Ethnicity**		
Brahmin	75	17.05
Chhetri	82	18.64
Madheshi	72	16.36
Janjati	177	40.22
Dalit	34	7.73
**Religion**		
Hindu	333	75.68
Buddhist	48	10.91
Muslim	6	1.36
Christian	53	12.05
**Place of residence**		
Village	227	62.95
City	163	37.05

^a^*NRs* Nepali Rupees

One hundred ninety-one (43.41%) patients suffered from CKD for less than 1 year, and 72.05% had developed end-stage of renal disease (stage V). A total of 67.27% participants were receiving hemodialysis services regularly, 58.11% had received hemodialysis for more than 1 year, and only 4.77% had received a transplanted kidney.

Three hundred fifty-six (80.91%) patients were taken to a hospital by their family members, 86.14% had received economic support from their family members for various medical expenses, and only 16.82% had health insurance. Two hundred ninety-six (67.27%) patients who were receiving hemodialysis services made regular visits to see a doctor at least 8–12 times per month. However, those who were not receiving hemodialysis services did not visit a doctor regularly. These patients only visited a doctor when they had actual health needs or for follow-up appointments but not on a fixed time interval.

Three hundred twenty-seven (74.32%) patients lived more than 30 km. away from the hospital where they were receiving treatments, and 35.22% paid 1001–5000 NPRs per visit; there were no fixed expenses during each visit. The minimum and maximum expenses were between 400 NPRs (3.49 US$) and 300,000 NPRs (2615.55 US$). Three hundred-seven (69.77%) patients were facing problems with their medical expenses (Table 2).

**Table 2:** Medical information and treatment experiences of participants

Characteristics	n	%
**Duration of illness** (years)		
< 1	191	43.41
1–5	151	34.32
> 5	98	22.27
**Stages of CKD**		
Stage I	89	20.22
Stage II	9	2.05
Stage III	7	1.59
Stage IV	18	4.09
Stage V	317	72.05
**Taking hemodialysis**		
Yes	296	67.27
No	144	32.73
**Duration of hemodialysis** (months)		
< 10	96	32.43
10–12	28	9.46
> 12	172	58.11
**History of kidney transplantation**		
Yes	21	4.77
No	419	95.23
**Medical conditions**		
***Hypertension***		
*Yes*	*404*	*91.82*
*No*	*36*	*8.18*
***Diabetes mellitus***		
*Yes*	*94*	*21.36*
*No*	*346*	*78.64*
***Heart disease***		
*Yes*	*50*	*11.36*
*No*	*390*	*88.64*
***Other chronic disease***		
*Yes*	*11*	*2.50*
*No*	*429*	*97.50*
***Disability***		
*Yes*	*5*	*1.14*
*No*	*435*	*98.86*
**Taking to a hospital**		
Family members	356	80.91
Friends	5	1.14
Themselves	79	17.95
**Frequency to see a doctor per month**		
Irregular	144	32.73
8	241	54.77
12	55	12.50
**Distance of hospital** (km)		
< 10	56	12.73
10–30	57	12.95
> 30	327	74.32
**Medical expenses per visit** (NRs)		
< 1001	185	42.05
1001-5000	155	35.22
5001-10,000	8	1.82
10,001-20,000	70	15.91
> 20,000	22	5.00
Min. = 400, Max. = 300,000, Median = 1500, IQR = 4000.00		
**Facing problem for medical expenses**		
Yes	307	69.77
No	133	30.23
**Support for medical expenses**		
Family members	379	86.14
Themselves	59	13.41
Others	2	0.45
**Having health insurance**		
Yes	74	16.82
No	366	83.18

In summary, the QOL level of participants in different domains was as follows: PCS: mean = 1093.50, SD = 591.68; MCS: mean = 744.32, SD = 341.06; KDCS while receiving hemodialysis: mean = 2218.08, SD = 894.25; and KDCS while not receiving hemodialysis: mean = 2744.54, SD = 340.54 (Table 3).

**Table 3:** Summary of scores of QOL of participants in different domains

Domains	Total	Mean (%)	SD	Min.	Max.
PCS	2100	1093.50 (52.07)	591.68	0	1950
MCS	1400	744.32 (53.16)	341.06	0	1380
KDCS taking hemodialysis^a^	4300	2218.08 (51.58)	894.25	423.32	3824
KDCS without hemodialysis^b^	4000	2744.54 (63.82)	340.54	1515.00	3380.00

^a^ Calculated based on the pooled of burden of kidney disease, symptoms/problem list, effects of kidney disease, work status, cognitive function, quality of social interaction, sleep, social support, dialysis staff encouragement and patient satisfaction

^b^ Calculated based on the pooled of burden of kidney disease, symptoms/problem list, effects of kidney disease, work status, cognitive function, quality of social interaction, sleep, social support

Two hundred thirty-six (53.64%) participants had good QOL in PCS, 22.05% had good QOL in MCS, 21.28% had good QOL in KDCS while receiving hemodialysis, and 13.19% had good QOL in KDCS while not receiving hemodialysis (Table 4).

**Table 4:** Summary of level of QOL in different domains

Domains	Total	Level of QOL					
		Poor (< Mean-1SD)		Moderate (Mean+/−1SD)		Good (> Mean + 1SD)	
		n	%	n	%	n	%
PCS	440	102	23.18	102	23.18	236	53.64
MCS	440	66	15.00	277	62.95	97	22.05
KDCS taking hemodialysis	296	58	19.59	175	59.12	63	21.28
KDCS without hemodialysis	144	21	14.58	104	72.22	19	13.19

In the univariate model, 19 variables were found to be associated with PCS-QOL: age, education, occupation, marital status, ethnicity, religion, duration of illness, present stage of CKD, receiving hemodialysis, duration of hemodialysis, history of kidney transplantation, being taken to a hospital, having health insurance, frequency of seeing a doctor per month, distance from the hospital, medical expenditure per visit, facing problems with medical expenses, diabetes mellitus and heart disease (Table 5). In the multivariate analysis, 8 variables were found to be associated with good PCS QOL. Those aged 31 to 50 years old had better QOL than those aged 51 years and over, with a 2.93-fold difference (95% CI = 1.48–5.76). Those who graduated from school at the tertiary level had better QOL than those who were illiterate (4.34-fold difference; 95% CI = 1.42–13.27). Those who were at stage I through stage IV had better QOL than those who were at stage V, with a 21.24-fold difference (95% CI = 2.97–151.77). Those who were not receiving hemodialysis had better QOL than those who were receiving hemodialysis (16.09-fold difference; 95% CI = 1.81–142.90). Those who traveled to a hospital to see a doctor by themselves had better QOL than those who were transported by their family members by a factor of 7.98 (95% CI = 3.65–17.40). Those who had their own health insurance had better QOL than those who did not (3.99-fold difference; 95% CI = 1.87–8.52). Those who spent 1001–5000 NPRs for medication per visit were less likely to have good QOL than those who spent less than 1000 NPRs (0.44-fold difference; 95% CI = 0.22–0.87). Those who were not facing problems with medical expenditures had better QOL than those who were facing problems by a factor of 2.19 (95% CI = 1.01–4.74) (Table 5).

**Table 5:** Factors associated with good-QOL in domain of PCS in univariate and multivariate analyses

Factors	OR	95%CI	p-value	OR_**Adj**_	95%CI	p-value
**Sex**						
Male	1.00					
Female	1.10	0.75–1.60	0.622			
**Age** (year)						
< 31	2.52	1.38–4.59	0.002^a^	1.68	0.71–3.99	0.233
31–50	2.68	1.74–4.14	< 0.001^a^	2.93	1.48–5.76	0.002^a^
> 50	1.00			1.00		
**Education**						
Illiterate	1.00			1.00		
Primary	1.11	0.64–1.92	0.701	1.56	0.66–3.67	0.304
Secondary	2.94	1.79–4.82	< 0.001^a^	1.91	0.81–4.51	0.136
Tertiary	2.61	1.36–4.98	0.004^a^	4.34	1.42–13.27	0.010^a^
**Occupation**						
Employed and retired	1.00					
Unemployed	1.81	1.09–3.01	0.020^a^			
**Annual household income** (NRs^b^)						
No income	1.00					
< 1,00,000	1.37	0.83–2.25	0.212			
≥ 1,00,000	1.49	0.88–2.55	0.136			
**Marital status**						
Single	4.29	1.86–9.90	0.001^a^			
Married	3.50	1.86–6.59	< 0.001^a^			
Widow/Divorced	1.00					
**Ethnicity**						
Brahmin	1.00					
Chhetri	1.03	0.54–1.97	0.912			
Madheshi	0.35	0.18–0.69	0.002^a^			
Janjati	0.80	0.46–1.38	0.428			
Dalit	0.44	0.19–1.00	0.052			
**Religion**						
Hindu	7.44	3.51–15.75	< 0.001^a^			
Buddhist	4.54	1.85–11.09	0.001^a^			
Christian	1.00					
**Place of residence**						
Village	1.00					
City	1.45	0.98–2.15	0.059			
**Duration of illness** (years)						
< 1	4.01	2.67–6.02	< 0.001^a^			
≥ 1	1.00					
**Stages of CKD**						
Stage I-IV	39.80	15.80–100.21	< 0.001^a^	21.24	2.97–151.77	0.002^a^
Stage V	1.00			1.00		
**Taking hemodialysis**						
Yes	1.00			1.00		
No	38.94	17.55–86.41	< 0.001^a^	16.09	1.81–142.90	0.013^a^
**Duration of hemodialysis** (months)						
< 10	2.25	1.31–3.87	0.003^a^			
10–12	8.25	3.37–20.15	< 0.001^a^			
> 12	1.00					
**History of kidney transplantation**						
Yes	8.84	2.03–38.44	0.004^a^			
No	1.00					
**Taking to hospital**						
Family members	1.00			1.00		
Themselves	5.20	2.86–9.44	< 0.001^a^	7.98	3.65–17.40	< 0.001^a^
**Support for medical expenses**						
Family members	1.00					
Themselves	1.02	0.59–1.75	0.938			
**Having health insurance**						
Yes	2.52	1.46–4.35	0.001^a^	3.99	1.87–8.52	< 0.001^a^
No	1.00			1.00		
**Frequency to see a doctor per month**						
Irregular	38.94	17.55–86.41	< 0.001^a^			
8–12	1.00					
**Distance of hospital** (km)						
< 10	1.00					
10–30	3.08	1.43–6.64	0.004^a^			
> 30	1.99	1.11–3.56	0.021^a^			
**Medical expenses per visit** (NRs^b^)						
< 1001	1.00			1.00		
1001-5000	1.24	0.80–1.90	0.329	0.44	0.22–0.87	0.019^a^
> 5000	13.20	6.44–27.01	< 0.001^a^	0.19	0.03–1.09	0.063
**Facing problem for medical expenses**						
Yes	1.00			1.00		
No	2.54	1.65–3.90	< 0.001^a^	2.19	1.01–4.74	0.045^a^
**Hypertension**						
Yes	1.00					
No	1.21	0.59–2.48	0.599			
**Diabetes mellitus**						
Yes	1.00					
No	2.38	1.47–3.85	< 0.001^a^			
**Heart disease**						
Yes	1.00					
No	4.29	2.16–8.54	< 0.001^a^			

^a^ Significant level at α = 0.05; ^b^*NRs* Nepali rupees

In the univariate model, 17 variables were found to be associated with MCS-QOL: age, education, occupation, ethnicity, religion, place of residence, duration of illness, present stage of CKD, receiving hemodialysis, being taken to a hospital, having health insurance, frequency of seeing a doctor per month, distance from the hospital, medical expenses per visit, facing problems with medical expenses, diabetes mellitus and heart disease (Table 6). Six variables were found to be associated with good MCS QOL in the multivariate model. Those who were living in a city had better QOL than those living in a village, with a 1.98-fold difference (95% CI = 1.05–3.72). Those who were at stage I through stage IV had better QOL than those who were at stage V, with a 28.33-fold difference (95% CI = 10.47–76.62). Those who traveled to a hospital to see a doctor by themselves had better QOL than those who were transported by their family members by a factor of 3.70 (95% CI = 1.83–7.51). Those who had their own health insurance had better QOL than those who did not, with a 5.34-fold difference 95% CI = 2.47–11.54). Those who spent more than 5000 NPRs for treatment per visit were less likely to have good QOL than those who spent less than 1000 NPRs by a factor of 0.23 (95% CI = 0.08–0.62). Those who were not facing problems paying for medical expenditure had better QOL than those who were facing expenditure problems, with a 2.66-fold difference (95% CI = 1.47–4.81) (Table 6).

**Table 6:** Factors associated with good-QOL in domain of MCS in univariate and multivariate analyses

Factors	OR	95%CI	***p***-value	OR_**adj**_	95%CI	***p***-value
**Sex**						
Male	1.00					
Female	0.63	0.40–1.02	0.061			
**Age** (year)						
< 31	1.19	0.58–2.44	0.625			
31–50	1.92	1.18–3.13	0.009^a^			
> 50	1.00					
**Education**						
Illiterate	1.00					
Primary	0.44	0.20–0.94	0.036^a^			
Secondary	1.40	0.80–2.45	0.228			
Tertiary	0.97	0.45–2.07	0.945			
**Occupation**						
Employed/retired	1.00					
Unemployed	2.03	1.00–4.12	0.049^a^			
**Annual household income** (NRs^b^)						
No income	1.00					
< 1,00,000	0.82	0.46–1.46	0.505			
≥ 1,00,000	0.72	0.38–1.35	0.310			
**Marital status**						
Single	1.10	0.44–2.73	0.824			
Married	0.87	0.44–1.71	0.697			
Widow/ Divorced	1.00					
**Ethnicity**						
Brahmin	1.00					
Chhetri	0.54	0.27–1.07	0.080			
Madheshi	0.33	0.15–0.73	0.006^a^			
Janjati	0.35	0.19–0.65	0.001^a^			
Dalit	0.36	0.13–0.97	0.045^a^			
**Religion**						
Hindu	6.17	1.87–20.28	0.003^a^			
Buddhist	1.33	0.28–6.26	0.716			
Christian	1.00					
**Place of residence**						
Village	1.00			1.00		
City	1.94	1.23–3.06	0.004^a^	1.98	1.05–3.72	0.033^a^
**Duration of illness** (years)						
< 1	2.93	1.84–4.69	< 0.001^a^			
≥ 1	1.00					
**Stages of CKD**						
Stage I-IV	4.42	2.74–7.13	< 0.001^a^	28.33	10.47–76.62	< 0.001^a^
Stage V	1.00			1.00		
**Taking hemodialysis**						
Yes	1.00			1.00		
No	3.73	2.33–5.97	< 0.001^a^	3.70	1.83–7.51	< 0.001^a^
**Duration of hemodialysis** (months)						
< 10	1.00					
10–12	2.00	0.84–4.73	0.114			
> 12	0.00	0.00	0.995			
**History of kidney transplantation**						
Yes	0.57	0.16–1.99	0.385			
No	1.00					
**Taking to a hospital**						
Family members	1.00					
Others (myself)/friends	3.00	1.74–5.19	< 0.001^a^			
**Support for medical expenses**						
Family members	1.00					
Myself/friends/others	0.70	0.35–1.42	0.332			
**Having health insurance**						
Yes	2.82	1.65–4.82	< 0.001^a^	5.34	2.47–11.54	< 0.001^a^
No	1.00			1.00		
**Frequency to see a doctor per month**						
Irregular	3.73	2.33–5.97	< 0.001^a^			
8–12	1.00					
**Distance of hospital** (km)						
< 10	1.00					
10–30	3.23	1.22–8.51	0.018^a^			
> 30	1.97	0.85–4.55	0.109			
**Medical expenses per visit** (NRs^b^)						
< 1001	1.00			1.00		
1001-5000	1.02	0.59–1.76	0.919	0.43	0.17–1.03	0.061
> 5000	2.01	1.15–3.52	0.014^a^	0.23	0.08–0.62	0.004^a^
**Facing problem for medical expenses**						
Yes	1.00			1.00		
No	2.80	1.76–4.48	< 0.001^a^	2.66	1.47–4.81	< 0.001^a^
**Hypertension**						
Yes	1.00					
No	1.18	0.53–2.65	0.675			
**Diabetes mellitus**						
Yes	1.00					
No	2.22	1.15–4.29	0.017^a^			
**Heart disease**						
Yes	1.00					
No	3.54	1.23–10.14	0.018^a^			

^a^ Significant level at α = 0.05; ^b^*NRs* Nepali rupees

## Discussion

Based on the Standard Kidney Disease Quality of Life Short Form (KDQOL-SF™ 1) [26], 53.64% of 440 CKD patients in Nepal had good QOL in the domain of PCS and 22.05% had good QOL in the domain of MCS. Several factors were associated with good QOL in the domain of PCS, such as age, education, stage of CKD, being taken to a hospital for hemodialysis, having health insurance, amount of medical expenses, and not facing problems with medical expenses. For the domain of MCS, six factors were associated with good QOL among CKD patients: resident area, stage of the disease, being taken to a hospital to a hospital, having health insurance, amount of medical expenses, and not facing a problem with medical expenses.

In our study, CKD patients who were younger had better QOL than those who were older in terms of PCS. This finding was consistent with a study conducted in Australia, which reported that CKD patients who were younger had significantly better QOL than those who were older [29]. This finding is consistent with a few studies performed in the State of Palestine [30, 31], which reported that older age was associated with poor HRQOL. A previous study in Nepal [32] also reported that being younger offered a better chance of good QOL than being older. Another study conducted in Nepal [33] reported that older age was associated with poor Health related quality of life (HRQOL).

Moreover, we found that those with higher education had better QOL than those with lower education in the domain of PCS. Several studies [34–36] have reported that the impact of having higher education is related to better QOL among CKD patients, particularly in the domain of PCS. This finding is consistent with a study conducted in the United States [37], which reported that higher and longer duration of education were associated with higher HRQOL scores. A study in the State of Palestine [31] also reported that a higher education level was associated with better QOL. Many studies [38, 39] have supported that education is correlated with good QOL among CKD patients. This finding was clearly supported by a multicenter study in China [40] that found that a higher education level led to better QOL among CKD patients. However, a study in Greece reported that education had no impact on physical and mental QOL scores [25]. On the other hand, a cross-sectional study in the State of Palestine [30] reported that no formal education was associated with poor HRQOL.

The present study also found that patients in the early stage of CKD had better QOL than patients in the late stage in terms of both PCS and MCS. This finding is consistent with a study conducted in Ghana [6], which reported that CKD patients who were in the early stage had significantly better QOL than those who were in the later stages. This finding is also consistent with a study in Indonesia [41], which reported that patients at the initial stages of CKD had better QOL than those in the end stages of CKD. A meta-analysis that used information from 109 articles reported that CKD patients who were in the end stage had poorer QOL than those who were in the early stage of the disease. Finally, a systematic literature review reported that CKD patients in the late stages had poorer QOL than CKD patients with in the early stages of the disease [42]. A study performed in the State of Palestine [30] and Greece [43] confirmed that the QOL of CKD patients was affected by the stage of the disease.

In our study, it was found that CKD patients in Nepal who were receiving hemodialysis treatment had better QOL than those who were not in domain of PCS. This finding is supported by a study in South Africa that reported that CKD patients who had received hemodialysis treatments had better QOL than those who did not [44]. A study in Korea also reported that CKD patients receiving hemodialysis had better QOL than those who did not, significantly at all stages of CKD [45]. A systematic review study in Malaysia showed that CKD patients who had a high family income and were able to access hemodialysis had better QOL than those who did not have insurance [46].

Having health insurance was another significant factor related to good QOL among CKD patients in Nepal. From our study, in terms of both PCS and MCS, patients who had health insurance had a better chance of obtaining access to medical services, particularly in clinics with nephrology service units. This finding is consistent with a study conducted in Germany that reported that the health insurance of individuals impacted various domains of QOL [47]. Additionally, a hospital-based cross-sectional study in Ethiopia clearly demonstrated that CKD patients who were able to buy health insurance had better QOL than those who could not [48]. There is no scientific evidence of a relationship between health insurance and QOL among CKD patients in Nepal.

Those who were paying less medical costs, including those with no problem paying for all their medical expenses, were more likely to have better QOL than those who had a large amount of medical expenses and faced problems paying them in terms of both PCS and MCS. This finding was clearly supported by a study conducted in Ethiopia, which reported that CKD patients who had no problem accessing medical services, particularly in a hemodialysis clinic, had better quality of life than those who did not have health insurance and had a financial problem regarding their access to health care [48]. Another study reported that family income was a significant predictor of good QOL among CKD patients [49]. A study in Thailand clearly demonstrated that family financial burden was a key factor in reducing QOL among CKD patients [50].

Moreover, those who were spending less for all their medical expenses were more likely to have better QOL than those who were spending more in the domains of PCS and MCS. This finding is consistent with a study conducted in the United States showing that an increase in the cost of medication lowers QOL in the domains of PCS and MCS [51]. However, there is no scientific evidence available on this aspect in Nepal. In addition, a single center study in Ghana reported that CKD patients who had a higher income had better QOL [6], which meant that any patients who could afford their medical expenses had better QOL.

Interestingly, in our study, it was found that those who could take themselves to a hospital had better QOL compared with those who needed someone to take them to a hospital in the domains of PCS and MCS. This might be because those who could take themselves to a hospital were healthier than those who needed a supporter, particularly for those who were living in a city. There is no scientific information available for this factor. This is the first investigation on the association between being taken to a hospital and having good QOL.

It was found that CKD patients living in cities had better QOL than those living in villages in terms of MCS. He, et al. [52] demonstrated that CKD patients living in a city and taking themselves to a hospital had better QOL than those who were living in remote areas and needed someone to take them to a hospital. This finding is consistent with a study conducted in the State of Palestine [30], and a study in Greece [43] reported that the residency location of CKD patients was a possible determinant of good HRQOL among CKD patients.

Participants in the study were selected from 4 tertiary-level hospitals in Nepal. Hence, it can be concluded that the participants in our study might be representative of all CKD patients in Nepal and that the results could be generalized to all CKD patients in Nepal, particularly in terms of the magnitude of the problem (prevalence). All the participants were interviewed after completing hemodialysis or meeting with a doctor that day, which improved the quality of the data to assess QOL among CKD patients [53]. No one refused to participate in the study. However, due to the number of questions in the KDQOL-SF™ section, some participants expressed boredom during the completion of the questionnaire, which might have impacted their answers. This was the only limitation found in the study.

## Conclusion

A large proportion of CKD patients in Nepal have good QOL in the domain of PCS, while others have poor QOL. Among CKD patients, 67.27% were undergoing hemodialysis free of charge in a hospital. Those who received hemodialysis had two times better QOL than those who were not receiving hemodialysis. Improving health care services, particularly in providing hemodialysis to all CKD patients who meet the criteria for this treatment, would improve the overall QOL among CKD patients in Nepal. However, patients are required to pay for other costs, such as medicine, travel, and food. To improve QOL among CKD patients in Nepal in terms of PCS and MCS, public health interventions should be implemented by focusing on those who are older, are at a late stage of CKD and have poor education, particularly regarding support for financial expenses and for providing a better means of taking them to a hospital. Moreover, CKD patients should be given a proper job that can produce an income capable of supporting them and their families, taking into account their health conditions and problems that arise with having CKD. The government or other health agencies should provide appropriate health insurance for people in Nepal because it would enable patients to have affordable access to medical services, especially for those who have CKD.

## Data Availability

Attached file.

## Abbreviations

CI: Class interval
CKD: Chronic kidney disease
ESRD: End stage renal disease
HRQOL: Health related quality of life
IOC: Item objective congruence
KDCS: Kidney disease component summary
KDQOL-SF: Kidney disease quality of life- short form
MCS: Mental component summary
NCD: Non- communicable disease
NRs: Nepali rupees
PCS: Physical component summary
QOL: Quality of life
SD: Standard deviation
SPSS: Statistical package for social sciences program
WHO: World Health Organization
